# Line-of-sight stability in unmanned aerial vehicle relays for hybrid free-space optical and visible light communication links under atmospheric effects

**DOI:** 10.1371/journal.pone.0343564

**Published:** 2026-03-11

**Authors:** Maha Sliti, Salman Ghafoor, Sarra Ayouni, Manel Mrabet, Lassaad Ben Ammar, Muhammad Ijaz

**Affiliations:** 1 University of Carthage, Higher School of Communication of Tunis (SUP’COM), LR11TIC04, Communication Networks and Security Research Lab. & LR11TIC02, Green and Smart Communication Systems Research Lab, Carthage, Tunisia; 2 School of Electrical Engineering and Computer Science (SEECS), National University of Sciences and Technology (NUST), H-12, Islamabad, Pakistan; 3 Department of Information Systems, College of Computer and Information Sciences, Princess Nourah bint Abdulrahman University, Riyadh, Saudi Arabia; 4 Department of Computer Sciences, College of Computer Engineering and Sciences, Prince Sattam bin Abdulaziz University, Al-Kharj, Saudi Arabia; 5 Department of Engineering, Faculty of Science and Engineering, Manchester Metropolitan University, Manchester, United Kingdom; Guangdong University of Petrochemical Technology, CHINA

## Abstract

Optical links supported by an unmanned aerial vehicle (UAV) must sustain gigabit-class throughput despite atmospheric attenuation, turbulence, and platform-induced pointing errors. Single-technology designs based on free-space optical (FSO) or visible light communication (VLC) often lack robustness under changing altitude and weather conditions. This paper proposes a hybrid FSO/VLC UAV relay and a unified analytical model that combines Beer–Lambert path loss, turbulence-induced scintillation, and pointing jitter, coupling an FSO backhaul to a Lambertian V1LC access channel with a finite receiver field of view (FOV). MATLAB-based results show that, under light desert dust (extinction $\kappa \approx 0.35\;km^{-1}$), the FSO branch retains ≈70.5% of the transmitted power at 1 km and ≈49.7% at 2 km, compared to ≈81.9% and ≈67.0% in clear visibility ($\kappa =0.2\;km^{-1}$) and ≈49.7% and ≈24.7% in haze ($\kappa =0.7\;km^{-1}$). The VLC branch maintains a signal-to-noise ratio (SNR) of at least 30 dB when $FOV\leq 20^{\circ }$ for altitudes below 150 m. For line-of-sight stability, with a receiver capture half-angle of 20 μrad, the alignment probability is ≈99.97% at a root-mean-square (RMS) pointing jitter of 5 μrad (stabilized) versus ≈58.9% at 15 μrad (weak/no-stabilization control). These results provide practical thresholds for robust hybrid relays, including visibility-aware switching between FSO and VLC and sub-10 μrad stabilization to maintain high alignment probability for smart-city and emergency scenarios.

## 1 Introduction

The rapid expansion of smart cities, Internet of Things (IoT) ecosystems, and mission-critical networks for disaster recovery is driving an urgent need for wireless links capable of delivering high data rates, ultra-low latency, and energy-efficient communication. Conventional radio-frequency (RF) technologies face significant limitations due to spectrum congestion, susceptibility to interference, and increasingly strict regulatory constraints in dense urban deployments. These challenges have accelerated the exploration of optical wireless communication (OWC) as a promising alternative, leveraging unlicensed optical spectrum, narrow beam divergence, and immunity to RF interference [1–5].

OWC includes both free-space optical (FSO) links and visible light communication (VLC) systems. FSO excels in long-range, high-capacity, and secure transmission, particularly for backhaul and point-to-point connectivity. However, it is highly sensitive to adverse weather (fog, haze, desert dust) and atmospheric turbulence, which induce attenuation, scattering, beam wander, and scintillation that can degrade link reliability [6–8]. Although multi-hop relaying and spatial diversity have been proposed to mitigate these impairments [9–11], performance remains strongly environment-dependent under rapidly varying atmospheric and mobility conditions [12–15]. Recent experiments also show that high-rate optical links can be maintained under fast tracking and mobility, including coherent FSO at low Earth orbit (LEO) tracking rates [16] and UAV-enabled high-speed retro-reflective FSO links [17], motivating explicit modeling of alignment constraints in aerial optical relays.

VLC provides short-range, high-speed connectivity using LED sources in the visible spectrum, making it a natural complement to FSO. Its performance is constrained by receiver field-of-view (FOV) limits, sensitivity to misalignment, and mobility-induced fading in dynamic aerial deployments [3,18]. These effects are amplified in unmanned aerial vehicle (UAV) architectures, where platform vibrations, drift, and fast motion increase outage risk. Drone-based VLC demonstrations for post-disaster monitoring further highlight the practical role of acquisition and mobility constraints in aerial optical access links [19].

UAVs enable on-demand airborne relays that can be rapidly deployed in infrastructure-limited, remote, or disaster-stricken areas. UAV-assisted FSO can exploit elevated line-of-sight (LoS) paths to improve connectivity where terrestrial links are obstructed. Nevertheless, UAV-mounted optical links face reliability challenges due to pointing errors, atmospheric fluctuations, and platform dynamics [20–24]. Prior work has explored optimized hovering strategies [25], learning-assisted tracking [3], secure routing [26], and adaptive coding/modulation [27]; nevertheless, these approaches typically address isolated aspects and do not provide a unified treatment that jointly captures atmospheric impairments and UAV-induced LoS instability within a hybrid optical framework.

In this work, we develop a unified analytical model for a hybrid FSO/VLC UAV relay and validate its design implications through MATLAB-based numerical evaluation under representative desert-visibility conditions. The atmospheric channel is modeled using standard, tractable components (Beer–Lambert extinction and Gamma–Gamma turbulence) with scenario-based parameters, rather than a fully predictive weather-coupled model. This choice enables transparent design insights and closed-form trends, while opportunities for integrated predictive models are discussed in the conclusion section.

### 1.1 Contributions

Hybrid FSO–VLC relays have recently emerged as a compelling approach, combining the benefits of long-range, high-capacity infrared FSO beams with the advantages of license-free, short-range, high-throughput VLC downlinks. Several architectures have been investigated, including cascaded RF/FSO/VLC links [28–30], triple-hop multi-technology topologies [25,31], and non-orthogonal multiple access (NOMA) enabled dual-hop systems [32]. NOMA is a multiuser access technique in which multiple users share the same time–frequency resource by superposition coding at the transmitter and successive interference cancellation at the receiver.

Beyond individual architectures, the most relevant prior work can be synthesized along three axes that directly determine UAV-assisted hybrid optical reliability: (i) extinction/turbulence processes that set the FSO link budget and fading statistics, (ii) pointing and alignment maintenance under platform motion (tracking/retro-reflective or stabilized links), and (iii) VLC access constraints driven by Lambertian emission and receiver FOV/coverage trade-offs. This synthesis motivates the unified joint modeling adopted in this paper and clarifies how existing studies typically emphasize only one or two of these axes at a time.

Although these hybrid designs improve coverage and adaptability, most previous work isolates individual impairments: turbulence-induced fading [33], spatial diversity for coherent FSO links [34], or mitigation of RF interference in hybrid backhaul [35] rather than treating their *joint* impact in a UAV-assisted hybrid FSO↔VLC relay. To the best of our knowledge, only a limited number of studies provide a unified analytical treatment that simultaneously incorporates Beer–Lambert atmospheric extinction (κ), Gamma–Gamma scintillation and Gaussian LOS-jitter alignment ($\sigma_{\theta },\;\Theta_{c}$), while consistently coupling these effects with a Lambertian/FOV VLC branch to obtain tractable hybrid-availability expressions and altitude-dependent design rules. Table 1 synthesizes representative studies and highlights the remaining gap this paper addresses: an integrated model leading to practical, κ-indexed guidelines that map measured visibility to effective ranges, FOV settings, and switching policies. Relative to the UAV fronthaul statistical model of Najafi et al. [36], which focuses on FSO turbulence and geometric/pointing effects in isolation, the present work extends the same class of models by embedding them in a dual-branch FSO↔VLC relay, deriving closed-form hybrid availability expressions in (19), and mapping these to κ-indexed deployment thresholds summarized in Table 6. For additional comparative context, the simulation section further contrasts the proposed hybrid relay against pure-FSO, pure-VLC, and RF baselines and includes benchmark checks against standard statistical FSO models (see Tables 3 and 4).

**Table 1:** Representative UAV-assisted optical/hybrid studies and modeling gaps.

Reference	System / architecture	Impairments modeled	Key gap relative to this work
Petkovic et al. [28]	RF–FSO–VLC cascade (no UAV)	Extinction (Beer–Lambert)	No turbulence or pointing jitter; no altitude dependence
Multi-hop mixed-technology studies [25,30,31]	Multi-hop/triple-hop hybrid backhaul (no UAV)	Extinction (Beer–Lambert)	No turbulence or pointing jitter; limited coupling to a VLC access branch
Deka and Anees [32]	Dual-hop NOMA (no UAV)	Extinction + turbulence/scintillation	No pointing jitter; no hybrid (FSO/VLC) availability viewpoint
Turbulence-focused FSO works [33,34]	FSO links (analytical/experimental, no UAV)	Turbulence/scintillation only	No joint extinction+turbulence+pointing model; no VLC branch
Mondal and Hossain [37]	UAV multi-hop RF–FSO in dynamic environments	Extinction + turbulence/scintillation	Limited pointing-error treatment; no VLC access branch
Shen et al. [38]	UAV-assisted FSO with angle-of-arrival (AoA) fluctuations	Turbulence/scintillation + AoA fluctuations	No VLC branch; scenario-specific constraints
Wu et al. [39]	UAV-assisted hybrid FSO/RF under weather variation	Extinction + turbulence (scenario-based)	No unified pointing-jitter model coupled to VLC access
Najafi et al. [36]	UAV-based FSO fronthaul (statistical modeling)	Turbulence + geometric/pointing effects	No VLC branch; lacks visibility-indexed switching / deployment thresholds
Walsh et al. [16]	Coherent FSO at low Earth orbit (LEO) tracking rates (experimental)	High-rate link under fast tracking dynamics	Not UAV relay; no VLC branch; no visibility-indexed hybrid switching
Quintana et al. [17]	High-speed retro-reflective FSO with UAV (experimental)	Mobility/tracking; practical UAV FSO feasibility	Not hybrid FSO/VLC; limited joint modeling with extinction/turbulence+VLC
Takano et al. [19]	VLC on LED-equipped drone for post-disaster monitoring (experimental)	VLC acquisition/mobility constraints; camera-based detection	Not hybrid FSO/VLC relay; no unified extinction/turbulence/pointing model
Pešek et al. [40]	Hybrid FSO/VLC last-mile/last-meter (demonstration)	Practical feasibility of hybrid optical branching	No UAV dynamics; no altitude/pointing-jitter coupling
**This work**	**UAV-assisted hybrid FSO** ↔ **VLC relay with altitude-aware switching**	**Extinction + turbulence (Gamma–Gamma) + pointing jitter (Rayleigh), coupled to Lambertian/FOV VLC**	**Unified model and practical design thresholds (visibility-aware switching; divergence/FOV/jitter limits)**

To address this research gap, we introduce a UAV-assisted hybrid optical relay with the following contributions:

- **Integrated analytical modeling.** We develop a tractable framework that jointly accounts for Beer–Lambert atmospheric extinction (κ), Gamma–Gamma turbulence, and the alignment probability induced by Gaussian line-of-sight pointing jitter ($\sigma_{\theta },\;\Theta_{c}$), and couples the free-space optical branch to a Lambertian/field-of-view visible-light communication branch. The model yields closed-form or semi-analytical expressions for outage and hybrid-link availability.
- **Visibility-indexed thresholds and switching.** Building on this framework, we derive visibility-indexed (i.e., κ-dependent) operating thresholds that map measured visibility to effective range and parameter choices, and we specify pre-emptive handover rules for FSO↔VLC mode selection as a function of geometry and channel state.
- **Altitude window and VLC trade-offs.** Under representative arid-climate profiles, we identify an altitude window (for a fixed ground offset D) that maximizes hybrid availability via range–altitude geometry r(h,D), and quantify the signal-to-noise-ratio versus field-of-view trade-space on the VLC branch, including its impact on footprint size and coverage.
- **Actionable engineering guidance.** We translate these results into UAV relay deployment guidelines, providing numerically validated ranges for beam divergence $\theta_{div}$, receiver field-of-view, acceptable pointing-jitter levels, and handover policies that maintain target bit-error rate and availability in desert-visibility conditions relevant to smart-city and emergency scenarios.

### 1.2 Paper organization

The remainder of the paper is organized as follows. Sect 2 introduces the unmanned-aerial-vehicle relay topology and the hybrid FSO-VLC switching logic. Sect 3 formalizes Beer–Lambert atmospheric extinction, Gamma–Gamma turbulence, Gaussian pointing-jitter alignment probability, and the Lambertian/field-of-view VLC channel, and derives closed-form outage and hybrid availability expressions. Sect 4 evaluates system performance through numerical results, including availability versus altitude and visibility, comparative analysis of FSO, VLC, and hybrid links with respect to distance, the joint impact of pointing jitter and extinction, and VLC signal-to-noise ratio as a function of field-of-view and altitude. Sect 5 consolidates these findings into actionable engineering thresholds for beam divergence, receiver field-of-view, pointing stability, and visibility-indexed switching policies. Sect 6 summarizes the main contributions and highlights practical implications for smart-city and emergency deployments.

## 2 Proposed architecture

**Fig 1:**
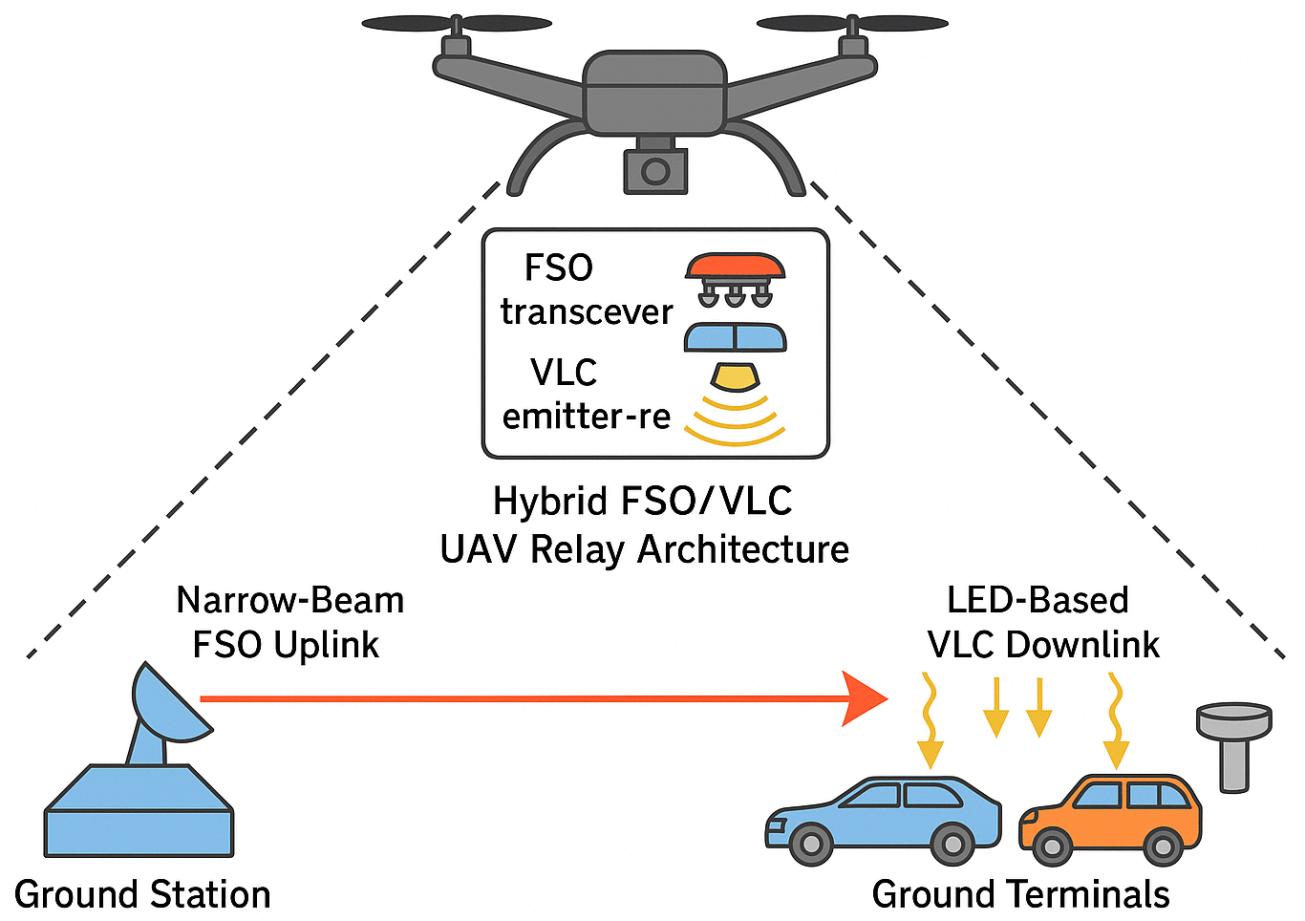
Hybrid FSO/VLC UAV relay architecture integrating narrow-beam FSO uplinks and LED-based VLC downlinks with dynamic link adaptation.

The proposed system is a UAV-assisted hybrid optical communication platform conceived to deliver high-speed, resilient links in situations where conventional infrastructure is limited, interrupted, or entirely absent. At its core is a dual-mode optical payload that combines a long-range FSO transceiver with a short-range VLC module. This combination allows the airborne relay to adjust in real time to atmospheric conditions and UAV-induced pointing variations, thereby maximizing link robustness. Fig 1 presents the overall design concept, illustrating the interaction between the airborne relay and the ground terminals.

The payload integrates two complementary optical subsystems. The FSO branch uses a 1550 nm laser diode, a collimator with sub-milliradian divergence, and a 50 mm aperture telescope to form a high-capacity, long-distance link with a ground station or terrestrial base station. This wavelength combines low atmospheric absorption with high eye safety, allowing gigabit-class data rates to be extended several kilometers in clear weather [20,21,41]. The VLC branch employs a high-power light-emitting diode (LED) array in the 450–650 nm spectrum to provide broadband, short-range connectivity to multiple users within the UAV coverage footprint. An adjustable concentrator lens photodiode with a field of view ranging from $15^{\circ }$ to $60^{\circ }$ supports wide-area illumination or high SNR-focused communication [1,18]. Both subsystems are mounted on a lightweight and stabilized gimbal with a fast-steering mirror for real-time beam alignment, which compensates for vibrations, wind-induced oscillations, and platform drift that otherwise would cause significant misalignment losses, particularly for the narrow FSO beam [20,34].

To sustain performance under varying conditions, the UAV relay continuously monitors atmospheric visibility (used to compute the extinction coefficient κ for Beer–Lambert loss), pointing stability (derived from inertial and optical feedback to estimate the LOS jitter $\sigma_{\theta }$ relative to the receiver capture half-angle $\Theta_{c}$), and instantaneous link metrics (FSO optical power and BER, VLC SNR). These measurements enable real-time adjustments within a dynamic operating framework. In clear and stable conditions (for example, $\kappa <0.3\;{km}^{-1}$ and $\sigma_{\theta }<10\;\mu rad$), the relay operates in an FSO dominant mode, achieving $BER<10^{-3}$ on distances of kilometer class [9,41]. Under reduced visibility or greater platform movement, the system shifts to VLC dominant operation, maintaining reliable short-range links with SNR>30dB for narrow FOVs at altitudes below 150 m [3,18]. When conditions fluctuate rapidly, both the FSO and VLC channels are used in parallel to provide redundancy and load balance [11,29]. An adaptive controller adjusts the beam divergence, power allocation, and modulation formats on–off keying (OOK)/pulse-position modulation (PPM) for FSO and OFDM for VLC based on real-time link quality indicators [27,35].

The ground network comprises a fixed base station equipped with high-power optical transceivers for the primary FSO uplink and mobile ground units equipped with VLC receivers for local access. Acting as an optical repeater, the UAV relays traffic with priority given to latency-sensitive and high-importance flows, such as real-time video and IoT sensor feeds. This configuration supports simultaneous transmission of VLC to multiple users while maintaining a secure narrow beam FSO backhaul for long-range connectivity [3,26].

This architecture offers inherent resilience to impairments: When weather-induced fading or pointing errors degrade the FSO path, the VLC maintains service at short range; conversely, when users move outside the high-SNR VLC zone, but visibility is favorable, the FSO link remains effective [6,9]. It also improves spectral efficiency and security, as narrow FSO beams limit interception risk and VLC operates in unlicensed optical bands immune to RF interference, with ambient light noise mitigated through optical filtering and FOV control [1,35]. Finally, UAV relays can be deployed in minutes, making the system highly flexible for temporary backhaul in emergencies, rapid expansion of smart city IoT coverage, or network deployment in challenging environments [20,25]. This combination of capabilities positions the design as a strong candidate for sixth-generation (6G) backhaul, rapid post-disaster restoration, and agile connectivity in dynamic urban or remote scenarios.

## 3 System model

**Fig 2:**
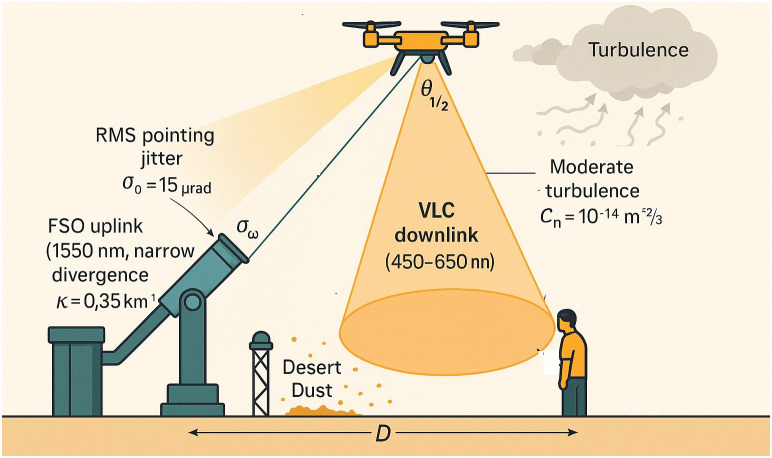
System model of a UAV-assisted hybrid optical link: a narrow-beam FSO uplink connects the ground terminal to the UAV, while a wide-aperture VLC downlink serves ground users.

In Fig 2, we model a hybrid UAV–assisted optical link that couples a narrow-beam FSO link with a VLC link (Throughout this section, the slant range r is expressed in meters unless otherwise stated. When Beer–Lambert attenuation with κ in $\begin{array}{c} {\text{km}}^{-1}\; \end{array}$ is used, we apply $r_{km}≜r/1000$ so that $\exp(-\kappa r_{km})$ is dimensionally consistent.). The UAV hovers at an altitude of h=120 m and carries (i) a 1550 nm FSO terminal of optical power $P_{t}=1$ W, beam divergence $\theta_{div}<1$ mrad, and a 50 mm telescope; and (ii) a 450–650 nm LED array with semi-angle at half-power $\Phi_{1/2}=15^{\circ }$. The operation targets light desert dust with extinction κ=0.35 $\begin{array}{c} {\text{km}}^{-1}\; \end{array}$ and moderate turbulence $\begin{array}{c} C_{n}^{2}=10^{-14}\; \end{array}$ m_−2/3_. The dynamics of the UAV induces Gaussian pointing jitter with RMS $\sigma_{\theta }=15\;\mu$rad. FSO employs OOK/PPM and VLC uses DC–biased OFDM, consistent with previous work [3,6,7,18,20,34].

### Parameter selection and representativeness.

The nominal extinction coefficient κ=0.35 $\begin{array}{c} {\text{km}}^{-1}\; \end{array}$ corresponds to light desert dust with meteorological visibility on the order of 10–12 km, consistent with desert FSO measurements and standard models in [6,7]. The strength of the turbulence $C_{n}^{2}=10^{-14}$ m_−2/3_ reflects moderate turbulence at night near the ground for horizontal paths of length 1–2 km [2]. The operational altitude h=120 m is chosen to match typical regulatory ceilings for small unmanned aircraft systems while ensuring that the FSO slant range spans 0–2 km for realistic ground offsets. Therefore, these parameter choices represent a concrete yet widely applicable desert-environment scenario rather than an artificially benign case.

### Atmospheric-model simplifications.

The atmospheric model is deliberately simplified to remain analytically tractable and to isolate first-order design trade-offs. Specifically, (i) the extinction coefficient κ is treated as *scenario-based and constant* over the link during each evaluation (no time-varying dust/fog dynamics), (ii) turbulence is summarized by a single $C_{n}^{2}$ value and mapped to Gamma–Gamma parameters via the Rytov variance (no altitude-dependent $C_{n}^{2}(z)$ profile, inner/outer-scale effects, or temporal correlation), and (iii) scattering microphysics and wavelength-dependent aerosol models are not explicitly simulated. Consequently, the reported thresholds should be interpreted as *engineering guidelines* under the assumed visibility and turbulence regimes rather than universal guarantees.

### Path loss and visibility.

The optical power received over a propagation path of length r is governed by the Beer–Lambert law:


$$P_{r}(r)=P_{t}\;T_{0}\;\exp\;(-\kappa r_{km}),$$ (1)

where $P_{t}$ denotes the transmitted optical power, $T_{0}$ the transmittance of the system, and κ ($\begin{array}{c} {\text{km}}^{-1}\; \end{array}$) the atmospheric extinction coefficient [42].

The sensitivity of the received power to variations in κ is obtained as follows:


$$\frac{\partial P_{r}}{\partial \kappa }=-r_{km}\;P_{r},\;r_{1/2}=\frac{\ln2}{\kappa },$$ (2)

indicating that an identical change Δκ penalizes longer transmission spans more severely. Moreover, the half-power distance $r_{1/2}$ scales inversely with the extinction coefficient (with $r_{1/2}$ expressed in km when κ is in $\begin{array}{c} {\text{km}}^{-1}\; \end{array}$).

When an estimate of visibility V (in km) is available, the standard extinction–visibility relationships summarized in [42] provide κ(V,λ), which can be substituted into Eq (1) to obtain power predictions dependent on visibility. A sensitivity threshold $P_{\text{th}}$ then determines the maximum usable range:


$$r_{\max}(\kappa ;P_{\text{th}})=\frac{1}{\kappa }\;\ln\;(\frac{P_{t}\;T_{0}}{P_{\text{th}}}),$$ (3)

where $r_{\max}$ is expressed in km when κ is in $\begin{array}{c} {\text{km}}^{-1}\; \end{array}$ (and can be converted to meters via $r_{\max,m}=1000\;r_{\max}$).

### Turbulence and fading statistics.

Atmospheric turbulence is quantified by the Rytov variance as:


$$\sigma_{R}^{2}=1.23\;C_{n}^{2}\;k^{7/6}\;r^{11/6},\;k=\frac{2\pi }{\lambda },$$ (4)

which characterizes the strength of turbulence-induced fluctuations along a path of length r (in meters) at wavelength λ. The corresponding scintillation index, valid in the weak-to-strong turbulence regimes [2], is given by:


$$\sigma_{I}^{2}=\exp\;\left(\frac{0.49\;\sigma_{R}^{2}}{(1+1.11\;\sigma_{R}^{12/5}{)}^{7/6}}+\frac{0.51\;\sigma_{R}^{2}}{(1+0.69\;\sigma_{R}^{12/5}{)}^{5/6}}\right)-1.$$ (5)

The probability density function (PDF) of the normalized received irradiance I is accurately modeled by the Gamma–Gamma distribution [43]:


$$f_{I}(I)=\frac{2(\alpha \beta {)}^{\frac{\alpha +\beta }{2}}}{\Gamma (\alpha )\;\Gamma (\beta )}\;I^{\frac{\alpha +\beta }{2}-1}\;K_{\alpha -\beta }\;\left(2\sqrt{\alpha \beta I}\right),\;I>0,$$ (6)

where Γ(·) denotes the Gamma function and $K_{\nu }(\cdot )$ is the modified Bessel function of the second kind.

The Gamma–Gamma shape parameters (α,β) are directly related to the Rytov variance through:


$$\alpha ={\left[\exp\;\left(\frac{0.49\;\sigma_{R}^{2}}{(1+1.11\;\sigma_{R}^{12/5}{)}^{7/6}}\right)-1\right]}^{-1},$$ (7)

$$\beta ={\left[\exp\;\left(\frac{0.51\;\sigma_{R}^{2}}{(1+0.69\;\sigma_{R}^{12/5}{)}^{5/6}}\right)-1\right]}^{-1}.$$ (8)

### Beam divergence and BER.

[44] The transmission divergence angle $\theta_{div}$ dictates the geometric coupling between the transmitted optical beam and the receiver aperture. For a narrow beam limited by diffraction that illuminates a receiver of area $A_{r}$ at a propagation distance r, the optical power received can be expressed as: [44]


$$P_{r}(r,\theta_{div})=P_{t}\;T_{0}\;\exp(-\kappa r_{km})\;\frac{A_{r}}{\pi r^{2}\theta_{div}^{2}},$$ (9)

implying that $P_{r}\propto \theta_{div}^{-2}$ for fixed r.

By mapping the received power to the detection signal-to-noise ratio (SNR) under intensity modulation with direct detection (IM/DD) and on–off keying (OOK) in additive white Gaussian noise (AWGN), the bit error rate (BER) can be approximated as


$${BER}_{\text{FSO}}(\theta_{div})\;\approx \;\frac{1}{2}\;erfc\;\left(\frac{\eta \;P_{r}(r,\theta_{div})}{2\;\sigma_{n}}\right),$$ (10)

where η denotes the effective responsivity (A/W), including modulation efficiency, and $\sigma_{n}^{2}$ is the post-detection noise variance.

To account for turbulence-induced fading, the average BER is obtained by expectation over the irradiance distribution $f_{I}(I)$:


$${\bar{P}}_{b,FSO}=\frac{1}{2}\int_{0}^{\infty }erfc\;\left(\frac{\eta \;P_{r}(r,\theta_{div})\;I}{2\sigma_{n}}\right)\;f_{I}(I)\;dI.$$ (11)

### Pointing jitter and alignment.

The residual jitter of the UAV is modeled as a zero-mean circular Gaussian process with angular RMS $\sigma_{\theta }$. With a receiver acceptance (capture) half-angle $\Theta_{c}$, the probability of two-dimensional radial alignment is expressed as:


$$P_{align}\;=\;1-\exp\;\left(-\frac{\Theta_{c}^{\;2}}{2\;\sigma_{\theta }^{\;2}}\right),$$ (12)

which can be tied to wind/actuation bandwidth via $\sigma_{\theta }=k_{v}\;v_{\text{wind}}$ for a given gimbal/fast-steering mirror (FSM) [20,34]. Pointing loss can be absorbed into $P_{r}$ or, equivalently, in the branch-outage definition below.

### VLC geometry and spatial SNR.

Consider a UAV directing its VLC source vertically downwards. The point of projection of the UAV, denoted by $P_{g}$, is defined as the intersection between the optical axis of the transmitter and the ground plane. For an arbitrary ground location (x,y), the link distance, the irradiance angle, and the incidence angle are given by


$$d(x,y)=\sqrt{h^{2}+x^{2}+y^{2}},\;\phi (x,y)=\arccos\;\;\;\left(\frac{h}{d(x,y)}\right),\;\psi (x,y)=\phi (x,y),$$ (13)

where h denotes the altitude of the UAV, d(x,y) the slant distance, ϕ(x,y) the angle of irradiance with respect to the transmitter optical axis, and ψ(x,y) the angle of incidence at the receiver.

The DC channel gain for a Lambertian source is [1]


$$H(0;x,y)=\frac{(m+1)A}{2\pi \;d(x,y{)}^{2}}\;\cos^{m}\;\phi (x,y)\;T_{s}\;g\;(\psi (x,y))\;\cos\psi (x,y),$$ (14)

where $m=-\ln2/\ln(\cos\Phi_{1/2})$, A is the detector area, $T_{s}$ the optical filter gain, and g(ψ) the concentrator gain within its acceptance. The footprint radius on the ground plane (at the UAV ground-projection point) is


$$r_{spot}\;=\;h\;\tan\Phi_{1/2}.$$ (15)

The spatial SNR field is then defined as follows:


$${SNR}_{VLC}(x,y)=\frac{(R\;P_{t}^{\left(VLC\right)}\;H(0;x,y){)}^{2}}{\sigma_{shot}^{2}+\sigma_{thermal}^{2}},$$ (16)

which we evaluate over the ground grid to determine coverage contours and rate maps.

### End–to–end error rates and availability.

For the VLC link, we consider:


$${BER}_{VLC}=Q\;(\sqrt{2\;{SNR}_{VLC}}),$$ (17)

consistent with IM/DD detection. Hybrid error rate under SNR thresholding on the FSO branch is as follows:


$$\begin{array}{cc} {BER}_{hybrid} & \;={BER}_{FSO}\cdot \left(1-P_{FSO\text{-}out}\right)+{BER}_{VLC}\cdot P_{FSO\text{-}out},\\ P_{FSO\text{-}out} & \;=\mathrm{Pr}\;\left({SNR}_{FSO}<\gamma_{th}\right). \end{array}$$ (18)

Finally, when branch outages explicitly include pointing and both branches share the same line-of-sight jitter, the dual–mode availability is


$$P_{avail}=1-P_{out},$$ (19a)

$$P_{out}=\int_{0}^{\infty }\;P_{out,FSO}(\gamma_{th}|J=j)\;P_{out,VLC}(\gamma_{th}|J=j)\;f_{J}(j)\;dj,$$ (19b)

where $P_{out}$ corresponds to the event that *both* branches are simultaneously in outage (i.e., neither mode is usable), and the conditional product form follows from conditional independence given the common jitter realization J=j.

The jitter magnitude is modeled as Rayleigh,


$$f_{J}(j)=\frac{j}{\sigma_{\theta }^{2}}\exp\;(-\frac{j^{2}}{2\sigma_{\theta }^{2}}),\;j\geq 0,$$ (20)

so that outages are conditionally independent *given* J=j but correlated unconditionally.

For branch b∈{FSO,VLC} with acceptance (capture) half-angle $\Theta_{c}^{\left(b\right)}$, the conditional branch outage decomposes into pointing and channel/turbulence effects as


$$\begin{array}{c} P_{out,b}(\gamma_{th}|J=j)=\left\{ \begin{array}{cc} P_{out,b}^{ch}(\gamma_{th};\;r(h),\ldots ), & j\leq \Theta_{c}^{\left(b\right)},\\ 1, & j>\Theta_{c}^{\left(b\right)}, \end{array}\right.\; \end{array}$$ (21)

where $P_{out,b}^{ch}$ denotes the channel/turbulence outage under alignment (computed from the received-power/SNR model for branch b).

For a fixed horizontal separation D, the FSO slant range is


$$r(h)=\sqrt{D^{2}+h^{2}}.$$ (22)

Substituting r(h) into (1) and (9), and evaluating (19) as a function of h, reveals an altitude window in which opposing trends in VLC footprint/SNR versus FSO path loss and pointing sensitivity maximize the overall availability.

## 4 Simulation results and discussion

### 4.1 Simulation parameters

We developed a MATLAB simulation of the optical payload of our hybrid UAV relay, which integrates a 1550 nm FSO transceiver (laser diode, <1 mrad collimator, 50 mm telescope) and a VLC module based on a 450–650 nm LED (semi-angle $\Phi_{1/2}=15^{\circ }$, concentrator photodiode). Path loss is computed using the Beer–Lambert law, turbulence-induced fading through the Rytov variance and Gamma–Gamma statistics, and LoS stability through a Gaussian pointing-jitter model ($\sigma_{\theta }=15\;\mu rad$). A desert search-and-rescue scenario uses an FSO uplink (1 W launch) to a UAV at 120 m under light dust (κ=0.35 $\begin{array}{c} {\text{km}}^{-1}\; \end{array}$) and $C_{n}^{2}=10^{-14}\;$m_−2/3_, with OOK/PPM and power adaptation that emulates scintillometer feedback [7,8]. The VLC downlink covers a ground-projection footprint of radius $r_{spot}=h\tan\Phi_{1/2}\approx 32$ m at 1 Gbps via DC-biased OFDM; a 200 Hz FSM compensates for jitter and target motion [3,18]. Table 2 lists all parameters.

**Table 2:** Simulation parameters.

**Environment**	
Terrain	Semi-arid desert
Extinction coefficient κ	$0.35\;km^{-1}$
Turbulence strength $C_{n}^{2}$	$1\times 10^{-14}\;m^{-2/3}$
**Geometry**	
Slant range r	0–2000 m
UAV altitude h	120 m
VLC footprint radius $r_{VLC}$	≈32 m (for $\Phi_{1/2}=15^{\circ }$)
**Optical components**	
FSO beam divergence $\theta_{div}$	0.8 mrad
VLC semi-angle at half power $\Phi_{1/2}$	$15^{\circ }$
Photodiode area A	$1\times 10^{-4}\;m^{2}$
Responsivity R	0.45 A/W
**Stabilization and noise**	
FSM bandwidth $B_{FSM}$	200 Hz
Pointing jitter RMS $\sigma_{\theta }$	15 μrad
Wind gust speed $v_{w}$	3 m/s
Electrical noise variance $\sigma_{n}^{2}$	$1\times 10^{-22}\;A^{2}$
VLC data rate $R_{VLC}$	1 Gbps
Observation time $T_{obs}$	10 s

In this section, $\kappa =0.35\;{km}^{-1}$ represents light desert dust and corresponds to a meteorological visibility on the order of 10–12 km using standard visibility–extinction relations κ(V,λ) (e.g., Kruse/Kim-type models). The turbulence parameter $C_{n}^{2}=10^{-14}\;m^{-2/3}$ is selected to represent a moderate near-ground nighttime regime over 1–2 km horizontal paths and we additionally evaluate sensitivity around this nominal value. Finally, the operational altitude h=120 m is chosen to match common small unmanned aircraft system (UAS) regulatory ceilings (e.g., 120 m above ground level (AGL) in the European open category and ≈400 ft AGL under U.S. Federal Aviation Administration (FAA) Part 107) while keeping the slant range within the considered 0–2 km window for realistic ground offsets.

### 4.2 Practical feasibility and hardware-limited parameters

This work is primarily a channel- and reliability-oriented evaluation of a hybrid FSO/VLC UAV relay. To ensure practical relevance, we explicitly relate the main physical-layer parameters to realistic payload and stabilization constraints.

#### Energy consumption and mission duration.

The optical transmit powers considered here (order of 1 W optical for the FSO uplink and a few-watt-class LED for the VLC downlink) are small compared with typical multi-rotor propulsion power (hundreds of watts). Therefore, the communication payload affects endurance mainly through added mass (which increases hover power) rather than through optical electrical power alone. In our scenario, the communication window ($T_{obs}=10$ s) represents a short burst within a longer mission (minutes), so the link results are compatible with practical UAV operating times.

#### Payload mass and stabilization overhead.

The proposed payload (50 mm receive telescope, compact laser/driver, and a VLC LED+photodiode front-end) is representative of small optical terminals suitable for small UAVs. The additional stabilization components (gimbal/FSM + tracking sensor) introduce both mass and electrical overhead; in this paper, their net effect on the link is captured by the residual pointing jitter parameter $\sigma_{\theta }$ (after stabilization) and by the receiver capture half-angle $\Theta_{c}$ (acceptance cone). We therefore interpret $\sigma_{\theta }$ as the *post-correction residual jitter* rather than raw airframe attitude motion.

#### FSM bandwidth and residual jitter consistency.

The assumed FSM closed-loop bandwidth ($B_{FSM}=200$ Hz) is intended to reject low-frequency components of UAV motion and target tracking error, while higher-frequency vibrations and unmodeled dynamics appear as residual jitter captured statistically by $\sigma_{\theta }$. To connect this to hardware limits, we include sensitivity sweeps over $\sigma_{\theta }$ and $\Theta_{c}$ (Sect 4.3), which directly quantify the availability loss when stabilization authority is reduced.

#### Hardware limits for $\theta_{0}\;$/acceptance angle.

In this study, $\theta_{0}$ (when used) corresponds to the receiver acceptance/capture half-angle $\Theta_{c}$, which is constrained by the optical front-end (telescope, tracking sensor FOV, and filtering). Larger $\Theta_{c}$ increases alignment robustness but may collect more background and relax optical selectivity; conversely, a smaller $\Theta_{c}$ improves selectivity but imposes stricter stabilization requirements. Our nominal values and sweeps are selected to reflect this practical trade-off and to provide engineering guidelines for feasible payload design.

### 4.3 Simulation results

**Fig 3:**
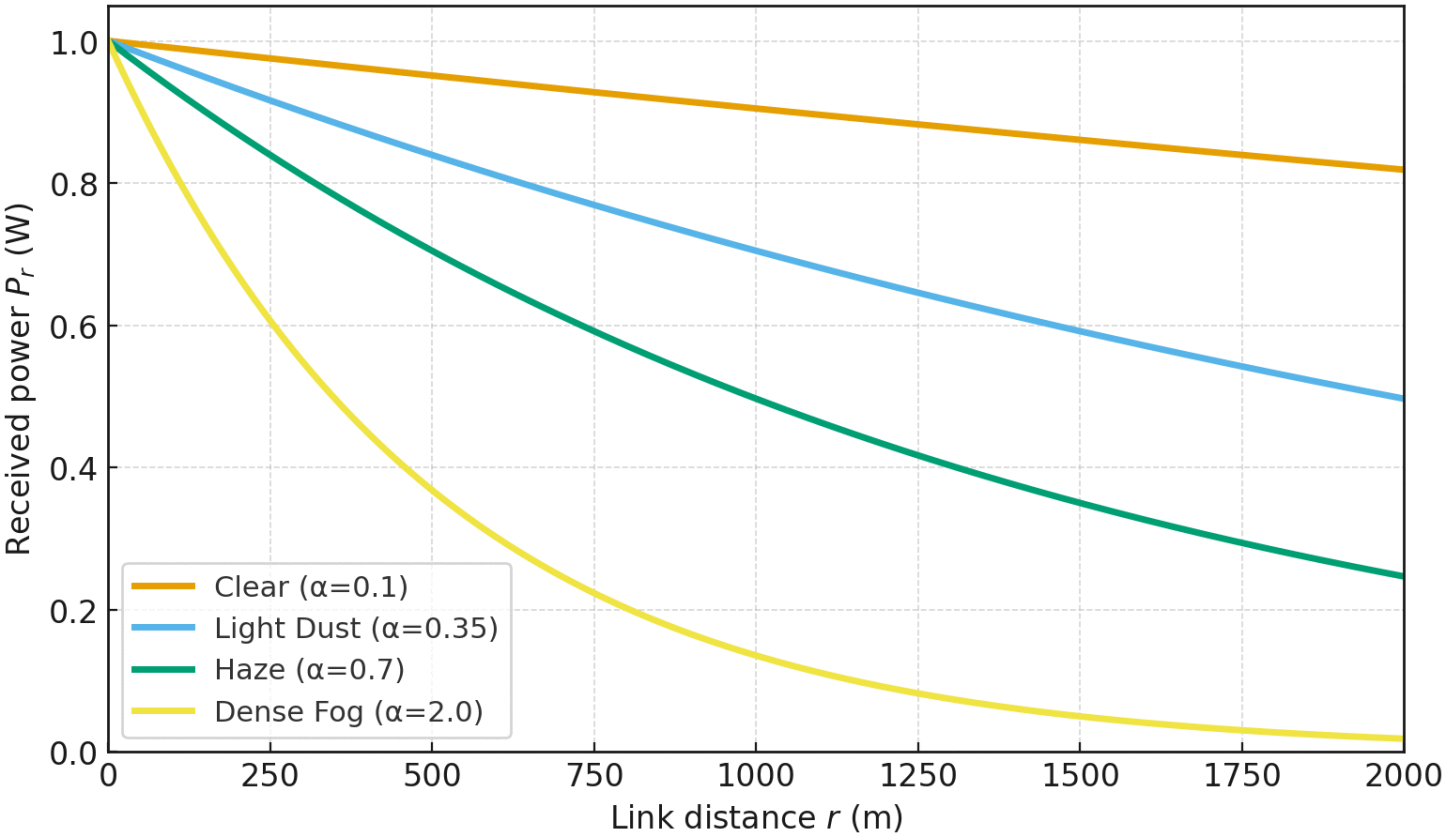
FSO received power versus link distance for varying visibility conditions (different extinction coefficients *κ*).

In Fig 3, the intersection points where the received power falls below critical thresholds shift dramatically with visibility: under haze (e.g., $\kappa \approx 0.7\;km^{-1}$) this point lies around 900 m, versus ~1600 m in light-dust conditions ($\kappa \approx 0.35\;km^{-1}$). By marking these “effective range” boundaries on the curve, one can derive a simple lookup table for system operators, allowing real-time link budgeting based on measured κ. Moreover, the exponential decay constant scales directly with κ, which quantifies how much additional optical power or aperture gain is required to compensate under low-visibility conditions.

**Fig 4:**
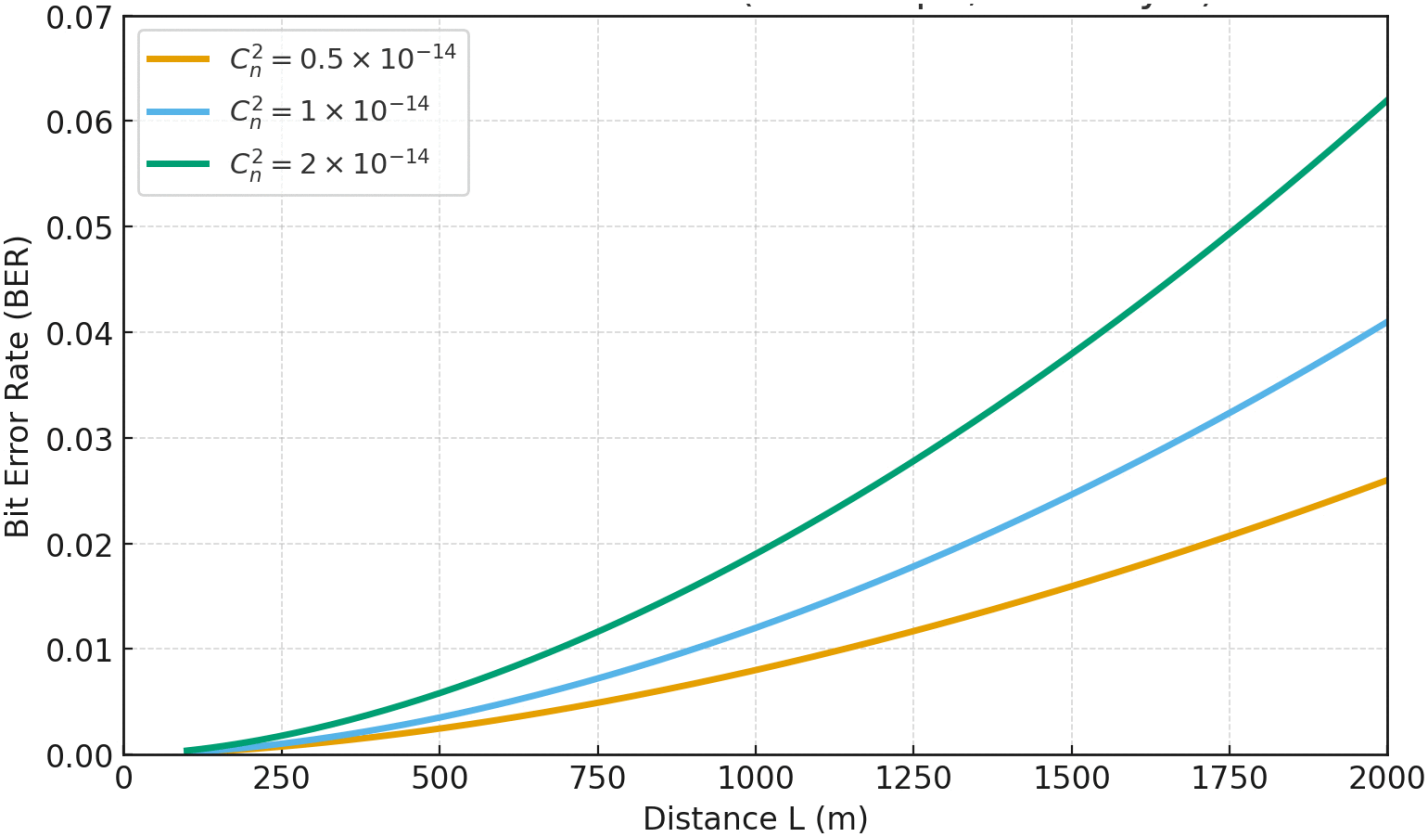
FSO bit-error rate (BER) versus link distance under different turbulence strengths (*C*^*2*^_*n*_).

Fig 4 shows that, with the noise floor tuned for a 10 dB SNR at 100 m, the FSO link maintains a BER below $10^{-3}$ up to approximately 300 m, but turbulence-induced fading drives the BER up to $10^{-2}$ by 1.5–2 km. This underscores that, while OOK transmission is highly reliable at short ranges, distances beyond 1 km require mitigation strategies, such as increased launch power, forward error correction coding, or a hybrid VLC/RF fallback, to preserve link integrity under moderate atmospheric turbulence. Moreover, the three curves corresponding to $C_{n}^{2}=\{0.5,1,2\}\times 10^{-14}\;m^{-2/3}$ illustrate how stronger turbulence markedly accelerates the degradation of BER with range. Adaptive optics or dynamic aperture control could further flatten these curves by compensating for beam wander. Finally, for applications that tolerate BER up to $10^{-3}$ (for example, environmental sensing), the FSO link is practical up to 1–1.5 km, beyond which hybrid architectures ensure continuous connectivity.

**Fig 5:**
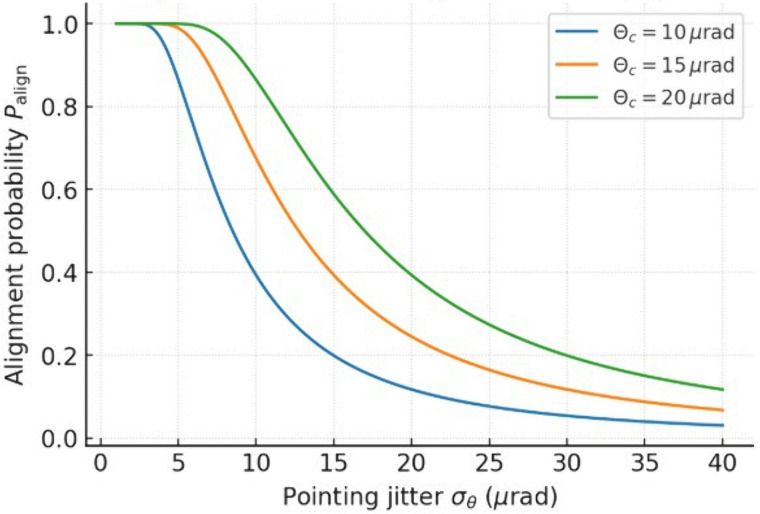
Line-of-sight (LoS) alignment probability versus RMS pointing jitter, for multiple receiver capture half-angles *Θ*_*c*_.

Fig 5 illustrates the relationship between RMS pointing jitter, $\sigma_{\theta }$, and the probability of maintaining line-of-sight (LoS) alignment, $P_{align}$, for three receiver capture half-angles $\Theta_{c}$ of 10 μrad, 15 μrad, and 20 μrad. As expected, larger capture angles result in greater tolerance to jitter, shifting the probability curves toward higher $\sigma_{\theta }$ values. For the narrowest acceptance angle ($\Theta_{c}=10\;\mu rad$), $P_{align}$ falls sharply, dropping below 50% at around 12 μrad jitter. Increasing $\Theta_{c}$ to 15 μrad delays this drop, maintaining $P_{align}>0.8$ up to approximately 12 μrad and above 0.5 until approximately 18 μrad. With $\Theta_{c}=20\;\mu rad$, the system maintains $P_{align}>0.9$ for $\sigma_{\theta }<10\;\mu rad$ and maintains $P_{align}$ above 0.5 until nearly 24 μrad. For an explicit control-group comparison supporting the “>99% alignment” conclusion, we evaluate the analytical expression of $P_{align}$ at a representative capture half-angle $\Theta_{c}=20\;\mu rad$. The stabilized case $\sigma_{\theta }=5\;\mu rad$ yields $P_{align}\approx 99.97\%$, whereas a weak/no-stabilization control case $\sigma_{\theta }=15\;\mu rad$ yields $P_{align}\approx 58.9\%$, quantifying the improvement achieved by tighter pointing control.

These results highlight the trade-off between optical receiver design and pointing control requirements. Increasing $\Theta_{c}$ relaxes the jitter tolerance, but can reduce optical gain and increase background noise in free-space optical links. Consequently, system designers must balance the acceptance angle with the divergence of the beam, the stability of the pointing, and the noise filtering to optimize link performance. For critical high-availability links, maintaining $\sigma_{\theta }<10\;\mu rad$ is advisable for narrow-beam systems, whereas wider capture angles can serve as a mitigation strategy against UAV-induced motion and wind disturbances.

**Fig 6:**
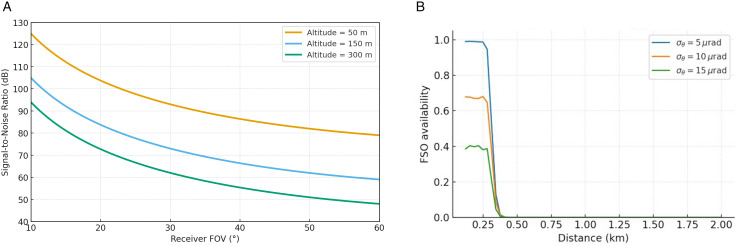
VLC SNR versus receiver field of view (FOV): (a) impact of altitude; (b) nominal operating altitude.

Fig 6(a) summarizes the VLC downlink signal-to-noise ratio (SNR) as a function of the receiver field of view (FOV) for multiple UAV altitudes, while Fig 6(b) highlights the nominal operating altitude used elsewhere in this work (h=120 m, $\Phi_{1/2}=15^{\circ }$). In all cases, the SNR decreases monotonically as the FOV widens, reflecting the fundamental trade-off between spatial coverage and optical gain: narrower FOVs concentrate more power on the photodiode (higher concentrator gain and lower collected background), whereas wider FOVs ease alignment and user mobility at the expense of reduced received signal strength.

**Fig 7:**
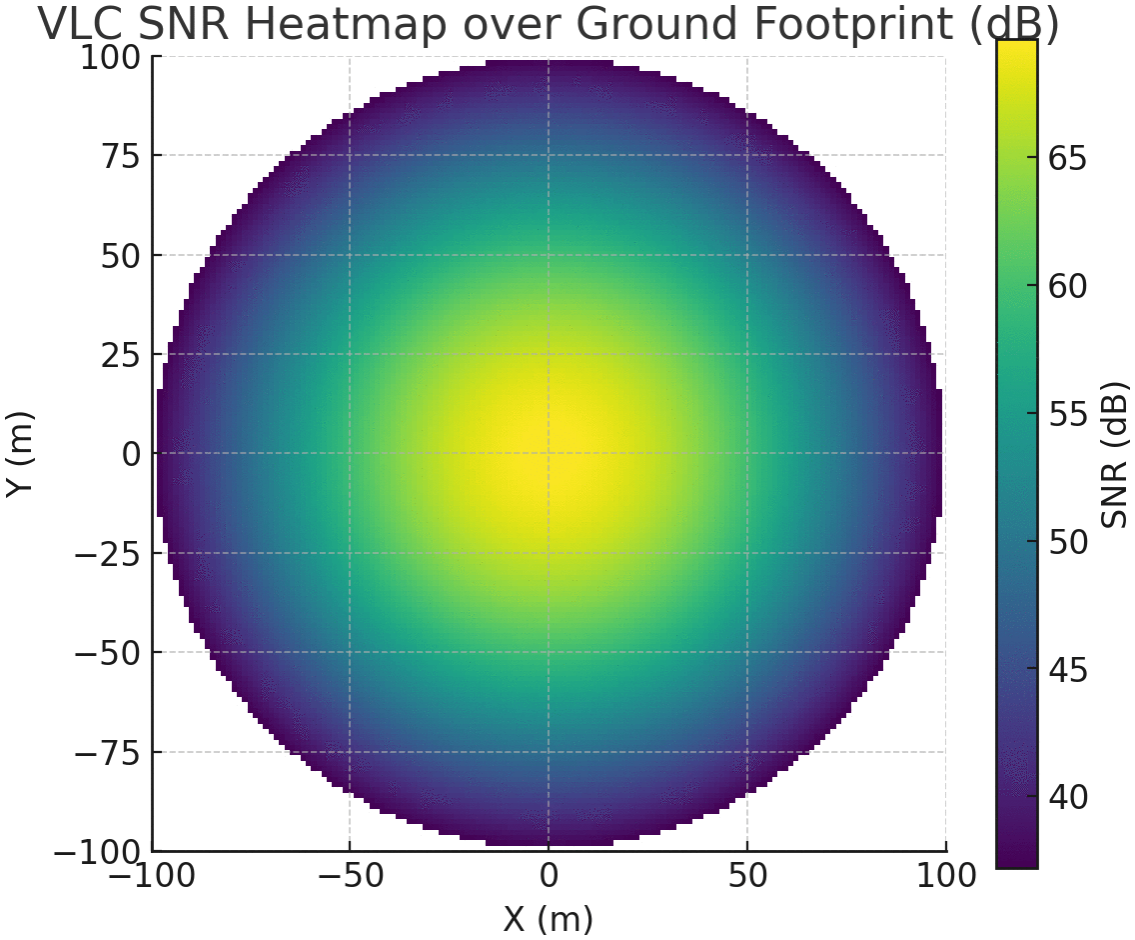
VLC SNR heatmap over the ground footprint at the nominal altitude and LED semi-angle at half-power.

As shown in Fig 7, at low altitude (h=50 m) the SNR exceeds 40 dB for $FOV\leq 30^{\circ }$ and remains around 30 dB even at $60^{\circ }$, indicating a robust link margin for 1 Gbps DC-biased OFDM. Increasing altitude shifts the curves downward due to larger path loss: at h=150 m, SNR ≥30 dB is maintained only for $FOV≲25^{\circ }$, while at h=300 m the SNR drops into the low-20 dB range as the FOV approaches $60^{\circ }$.

For the nominal altitude in Fig 6(b), the curve indicates an SNR of approximately 30 dB at $FOV=20^{\circ }$, decreasing to about 20 dB at $FOV=60^{\circ }$. Therefore, for high-speed and low-error operation (e.g., SNR≥25 dB for 1 Gbps OFDM), an FOV not greater than $\approx 30^{\circ }$ is recommended around h≈120 m, whereas deployments at h≥150 m should employ tighter receiver optics (e.g., $FOV\leq 20^{\circ }$) and/or adaptive beam steering. In scenarios requiring broader angular coverage (e.g., rapidly moving ground users), a multi-photodiode receiver or dynamic beam steering can preserve SNR across a wider effective FOV without sacrificing link reliability.

In Fig 7, the SNR gradient is remarkably shallow within 50 m of the UAV ground-projection point, remaining within 5 dB of its peak. This implies that small horizontal displacements of ground users incur only marginal performance penalties. Beyond 60 m, the SNR roll-off becomes steeper, decreasing by roughly 10 dB for each additional 20 m. This behavior is consistent with the dependence on the inverse-square distance in the Lambertian model and the $\cos^{m}$ gain term in (14). Consequently, to maintain an SNR above 30 dB for reliable 1 giga bit s^-1^ OFDM, the effective coverage radius should not exceed 60 m unless the LED optical power is increased or the receiver FOV is narrowed. Finally, the near-circular symmetry of the heatmap indicates that azimuthal misalignment has a minor impact compared with range, supporting the use of a fixed-radius planning model for user deployment.

**Fig 8:**
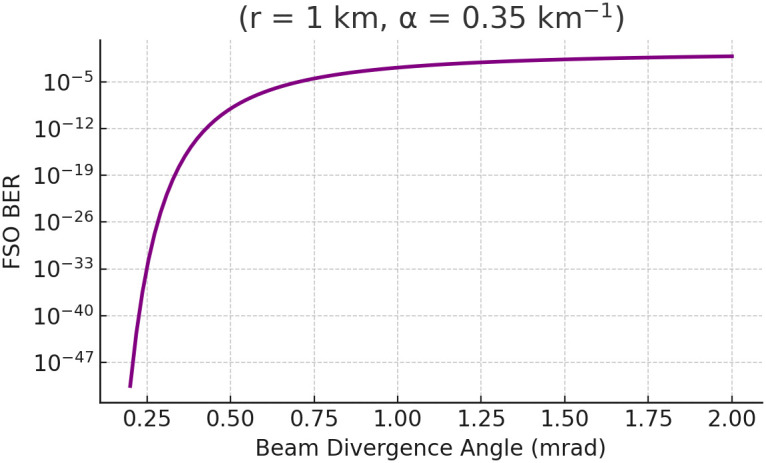
FSO BER versus beam divergence angle for a fixed range/channel/noise setting (illustrating divergence-driven misalignment trade-offs).

Fig 8 presents a sharp knee around 0.5–1 mrad: reducing divergence below this range produces diminishing returns in BER improvement, while beyond 1 mrad the BER degrades precipitously, increasing by more than two orders of magnitude for each additional 0.5 mrad. This inflection point can be used to set engineering specifications for the gimbal and FSM systems: if mechanical stabilization can only guarantee ±1 μrad jitter, then maintaining beam divergence at or below 0.8 mrad will help ensure that the BER remains below $10^{-5}$. In contrast, in applications with coarser pointing control, selecting a beam divergence above 1.2 mrad may be necessary to avoid catastrophic link outages despite the higher BER.

#### Baseline comparison (pure FSO, pure VLC, and RF relay).

To address the need for comparative baselines, we benchmark the proposed hybrid architecture against the baseline systems summarized in Table 3: (i) a *pure FSO* relay that relies only on the 1550 nm backhaul model and is declared available when $BER\leq 10^{-3}$, (ii) a *pure VLC* relay that relies only on the Lambertian downlink model and is declared available when SNR≥30 dB, and (iii) a *conventional RF relay* baseline using a standard large-scale path-loss model with a conservative SNR threshold (10 dB) over the same slant range. The hybrid system is declared available when *either* FSO (BER target met) *or* VLC (SNR target met) is available, consistent with the visibility-aware switching principle described earlier.

**Table 3:** Baseline systems used for comparative evaluation.

System	Availability criterion	Impairments captured
Pure FSO	$BER\leq 10^{-3}$	κ, turbulence ($C_{n}^{2}$), pointing jitter
Pure VLC	SNR≥30 dB	Lambertian loss, FOV, background noise
RF relay (baseline)	SNR≥10 dB	large-scale path loss (conservative)
Hybrid FSO/VLC (proposed)	FSO OR VLC criterion met	selects best link per conditions

#### Benchmark with previously published FSO and hybrid results.

To provide at least one benchmark against previously published work, we explicitly relate our modeling and key numerical operating points to canonical and peer-reviewed baselines, as summarized in Table 4. On the FSO side, the backhaul impairment set (Beer–Lambert attenuation with turbulence and pointing jitter) follows standard formulations used in the FSO literature with pointing errors and UAV-assisted FSO links [41,45]; accordingly, our received-power retention and alignment-probability trends match the expected exponential visibility dependence and the rapid degradation with increasing jitter reported in these references. On the VLC side, the access-channel modeling is based on the widely adopted Lambertian DC gain framework for LED VLC links [1,46], and our SNR–FOV behavior reproduces the well-known monotonic SNR decrease with increasing FOV due to concentrator-gain reduction. Finally, to position the proposed hybrid architecture within the broader hybrid relaying literature, we note that the considered two-hop structure and availability-driven link operation are consistent with previous hybrid relaying frameworks that cascade optical and VLC components [47].

**Table 4:** Minimal benchmark check versus standard published baseline models: FSO with pointing errors [41,45] and Lambertian VLC [1,46].

Item	Operating point in this work	Considered Value
FSO Beer–Lambert retention	$\kappa =0.35\;km^{-1}$, r=1 km	$P_{r}/P_{t}\approx 0.705$ (Fig 3)
FSO Beer–Lambert retention	$\kappa =0.35\;km^{-1}$, r=2 km	$P_{r}/P_{t}\approx 0.497$ (Fig 3)
Pointing benchmark (jitter sensitivity)	$\Theta_{c}=20\;\mu rad$, $\sigma_{\theta }=5\;\mu rad$	$P_{align}>0.99$ (Fig 5)
VLC Lambertian benchmark (FOV trade-off)	h=120 m, $\Phi_{1/2}=15^{\circ }$	SNR decreases with FOV (Fig 6)

#### Uncertainty and sensitivity analysis.

To quantify the robustness of the proposed hybrid relay to environmental and design uncertainty, we performed a parametric sensitivity study over (i) the extinction coefficient κ, (ii) the FSO beam divergence $\theta_{div}$ (denoted $\theta_{0}$ in some literature), and (iii) the VLC LED semi-angle at half power $\Phi_{1/2}$. Unless otherwise stated, all remaining parameters are fixed to the nominal values in Table 2. The swept sets, summarized in Table 5, are $\kappa \in \{0.2,0.35,0.7\}\;km^{-1}$, $\theta_{div}\in \{0.5,0.8,1.2\}\;mrad$, and $\Phi_{1/2}\in \{10^{\circ },15^{\circ },30^{\circ }\}$, which cover clear-to-hazy visibility and practical optical payload configurations.

**Table 5:** Sensitivity study settings.

Extinction κ (km_−1_)	{0.2, 0.35, 0.7}
FSO divergence $\theta_{div}$ (mrad)	{0.5, 0.8, 1.2}
VLC semi-angle $\Phi_{1/2}$ (deg)	{10, 15, 30}

## 5 Design guidelines

The following thresholds are distilled directly from Sect 4: (i) *visibility-indexed switching* near $\kappa \;\approx \;0.5\;km^{-1}$ at r≈1.5 km to preserve BER targets (cf. Figs 3–8); (ii) an *effective range* of ~1.6 km under $\kappa \;\approx \;0.35\;km^{-1}$ and ~0.9 km under $\kappa \;\approx \;0.7\;km^{-1}$ (Fig 3); (iii) a *divergence knee* at 0.5–1 mrad with a safe choice $\theta_{div}\leq 0.8$ mrad for $BER<10^{-5}$ (Fig 8); (iv) *VLC planning*: $FOV\leq 20^{\circ }$ below h≤150 m keeps SNR >30 dB with an effective coverage radius ≲60 m (Figs 6 and 7); and (v) *pointing stability*: targeting $\sigma_{\theta }<10\;\mu$rad yields high alignment probability under the Rayleigh jitter model in (20) (and the corresponding alignment relation in (12)).

Table 6 summarizes the recommended operating parameters derived from our analyses. For instance, maintaining a beam divergence $\theta_{div}\leq 0.8$ mrad ensures $BER<10^{-5}$ at 1 km; a receiver field of view $FOV\leq 20^{\circ }$ at h=150 m maintains SNR >30 dB; and RMS jitter $\sigma_{\theta }<10\;\mu$rad yields $P_{align}>0.9$. These thresholds can guide the design of UAV-mounted optics, gimbals, and link-adaptation algorithms.

**Table 6:** Recommended parameter settings for robust hybrid FSO/VLC relay.

Parameter	Recommended range	Rationale
Beam divergence $\theta_{div}$	≤0.8 mrad	Keeps $BER<10^{-5}$ at 1 km
Receiver FOV	$\leq 20^{\circ }$ (at h≤150 m)	SNR >30 dB for 1 Gbps
Pointing jitter $\sigma_{\theta }$	≤10 μrad	Alignment probability >90%
Switch threshold $\kappa_{th}$	$\approx 0.5\;km^{-1}$ at r=1.5 km	Pre-emptive FSO-to-VLC switching
Altitude	100–180 m (for κ=0.35)	Maximizes hybrid availability

## 6 Conclusion

This paper presented a UAV-assisted hybrid optical relay that combines FSO backhaul with VLC access to deliver high-speed and resilient airborne connectivity when terrestrial infrastructure is limited or disrupted. Taking advantage of the complementary strengths of narrow-beam infrared FSO links and short-range LED-based VLC coverage, the proposed architecture improves robustness under changing altitude, visibility, and platform stability.

We developed a unified analytical framework that captures the dominant impairments of airborne optical links, including Beer–Lambert attenuation governed by the extinction coefficient κ, turbulence-induced scintillation characterized through the Rytov variance and Gamma–Gamma fading, and LOS misalignment due to UAV motion modeled via a statistical pointing-jitter process. The framework was evaluated through MATLAB-based numerical simulations under representative desert-visibility conditions, using practical parameter values for $C_{n}^{2}$, receiver field of view, and RMS pointing jitter $\sigma_{\theta }$.

The results indicate that, under light dust, the FSO branch maintains strong received power and low error rates over short to medium ranges, while performance degrades at longer distances due to exponential attenuation and turbulence fluctuations. The VLC branch provides reliable short-range service within the UAV footprint, but its signal-to-noise ratio depends strongly on altitude and receiver FOV: narrow FOV settings offer high link margins for gigabit-class downlinks, whereas wider FOV increases mobility tolerance at the expense of optical gain. Pointing stability emerges as a critical requirement for both branches, with a marked reduction in alignment probability as $\sigma_{\theta }$ rises into the tens-of-microradian range, motivating fast steering and stabilization.

By using both optical technologies in a coordinated manner, the hybrid relay increases end-to-end availability compared with a standalone FSO link, since the VLC branch can sustain service during FSO degradations caused by reduced visibility, turbulence bursts, or transient pointing errors. The derived thresholds in terms of κ, altitude, FOV, divergence, and $\sigma_{\theta }$ translate these observations into practical guidance for configuring UAV payloads and adaptation policies in smart-city extensions, emergency response, and temporary backhaul deployments.

This study is simulation-based and relies on a deliberately tractable atmospheric model with scenario-defined κ and $C_{n}^{2}$ values that are held constant within each evaluation; therefore, it does not capture fast visibility fluctuations, altitude-dependent turbulence profiles, or correlated spatio-temporal channel dynamics. Future work will address these aspects by integrating atmosphere-aware predictive control that combines onboard visibility sensing (to infer κ), turbulence-strength estimation (to infer $C_{n}^{2}$), and data-driven or physics-informed forecasting to enable proactive switching and link adaptation. We will also extend the framework to multi-UAV cooperative relays and validate the proposed design through field experiments across diverse environments and weather conditions.

## Nomenclature

**Table:** 

Symbol	Definition	Units
*Geometry and kinematics*		
h	UAV altitude (vertical distance between UAV and ground plane)	m
(x,y)	Ground coordinates relative to UAV ground-projection point $P_{g}$	m
$P_{g}$	Ground-projection point of the UAV (intersection of beam axis with ground)	–
d(x,y)	Slant range from UAV at (0,0,h) to ground point (x,y,0)	m
ϕ(x,y)	Irradiance angle at the transmitter (w.r.t. boresight)	rad
ψ(x,y)	Incidence angle at the receiver (w.r.t. receiver normal)	rad
$\theta_{div}$	FSO transmit beam divergence (half-angle; see text for convention)	rad
$\Phi_{1/2}$	VLC LED semi-angle at half-power	deg
FOV	Receiver field-of-view half-angle (VLC branch)	deg
$\Theta_{c}$	Acceptance (capture) half-angle at the receiver (alignment cone)	rad
$A_{r}$	Receiver aperture/active area	m_2_
r	Propagation range along the optical path	m or $\begin{array}{c} {\text{km}}^{*}\; \end{array}$
*Propagation and turbulence*		
κ	Extinction (attenuation) coefficient (Beer–Lambert)	$\begin{array}{c} {\text{km}}^{-1} \end{array}$*
V	Meteorological visibility (used to infer κ(V,λ))	km
$C_{n}^{2}$	Refractive-index structure parameter	m−2/3
k	Optical wavenumber, k=2π/λ	$\begin{array}{c} {\text{m}}^{-1}\; \end{array}$
λ	Optical wavelength	m
$\sigma_{R}^{2}$	Rytov variance (turbulence strength)	–
$\sigma_{I}^{2}$	Scintillation index (normalized irradiance variance)	–
*Irradiance statistics (Gamma–Gamma model)*		
I	Normalized received irradiance	–
$f_{I}(I)$	PDF of irradiance (Gamma–Gamma)	–
α,β	Gamma–Gamma shape parameters (large-/small-scale turbulence)	–
Γ(·)	Gamma function	–
$K_{\nu }(\cdot )$	Modified Bessel function of the second kind	–
*Power, detection, and BER*		
$P_{t}$	Transmit optical power	W
$P_{r}$	Received optical power	W
$T_{0}$	System transmittance (optics + pointing losses not otherwise modeled)	–
η	Effective responsivity (incl. modulation scaling)	A/W
$\sigma_{n}^{2}$	Post-detection noise variance	A_2_
SNR	Electrical signal-to-noise ratio at detector output	–
BER	Bit-error rate (instantaneous)	–
${\bar{P}}_{b,FSO}$	Average BER over turbulence	–
*Alignment, jitter, and availability*		
$\sigma_{\theta }$	RMS line-of-sight jitter (zero-mean circular Gaussian model)	rad
$P_{align}$	Radial alignment probability within $\Theta_{c}$	–
$r_{1/2}$	Half-power distance ($P_{r}(r_{1/2})=\frac{1}{2}P_{t}T_{0}$)	m or km*
$r_{\max}$	Maximum usable range for threshold $P_{th}$	m or km*
$P_{th}$	Sensitivity/detection power threshold	W
*VLC branch (Lambertian)*		
m	Lambertian order, $m=-\ln2/\ln(\cos\Phi_{1/2})$	–
	(used in VLC channel gain and irradiance terms)	

## Supporting information

S1 Data VLC FSO Data. (ZIP)
